# Overview of Ankle Arthropathy in Hereditary Hemochromatosis

**DOI:** 10.3390/medsci11030051

**Published:** 2023-08-15

**Authors:** Sara Calori, Chiara Comisi, Antonio Mascio, Camillo Fulchignoni, Elisabetta Pataia, Giulio Maccauro, Tommaso Greco, Carlo Perisano

**Affiliations:** Orthopedics and Trauma Surgery Unit, Department of Aging, Orthopedic and Rheumatologic Sciences, Fondazione Policlinico Universitario Agostino Gemelli IRCCS, 00168 Rome, Italy; calorisara94@gmail.com (S.C.); chiara.comisi22@gmail.com (C.C.); antonio.mascio87@gmail.com (A.M.); camillo.fulchignoni@gmail.com (C.F.); elisabetta.pataia@policlinicogemelli.it (E.P.); giulio.maccauro@policlinicogemelli.it (G.M.); carlo.perisano@policlinicogemelli.it (C.P.)

**Keywords:** ankle joint, foot disease, hemochromatosis, arthropathy, total ankle arthroplasty, ankle arthrodesis

## Abstract

Hereditary hemochromatosis (HH) is an autosomal recessive bleeding disorder characterized by tissue overload of iron. Clinical systemic manifestations in HH include liver disease, cardiomyopathy, skin pigmentation, diabetes mellitus, erectile dysfunction, hypothyroidism, and arthropathy. Arthropathy with joint pain is frequently reported at diagnosis and mainly involves the metacarpophalangeal and ankle joints, and more rarely, the hip and knee. Symptoms in ankle joints are in most cases non-specific, and they can range from pain and swelling of the ankle to deformities and joint destruction. Furthermore, the main radiological signs do not differ from those of primary osteoarthritis (OA). Limited data are available in the literature regarding treatment; surgery seems to be the gold standard for ankle arthropathy in HH. Pharmacological treatments used to maintain iron homeostasis can also be undertaken to prevent the arthropathy, but conclusive data are not yet available. This review aimed to assess the ankle arthropathy in the context of HH, including all its aspects: epidemiology, physiopathology, clinical and imaging presentation, and all the treatments available to the current state of knowledge.

## 1. Introduction

Hereditary hemochromatosis (HH) is a recessive autosomal disorder of iron metabolism, in which an excessive intestinal absorption of iron leads to tissue damage in the liver, pancreas, joints, heart, and skin [1]. The most common forms of HH are due to inherited mutations in the HFE gene, which is responsible for the production of both the hepcidin protein and the human homeostatic iron regulator protein (HFE protein), which controls iron levels in hepatic cells [2].

Hepcidin is the primary regulator of dietary iron absorption binding to the iron transport protein (ferroportin) in enterocytes, preventing the release of absorbed iron into circulation and inhibiting the systemic release of iron from recycled erythrocytes [3]. Clinical manifestations of pathologic iron accumulation include liver disease, skin pigmentation, diabetes mellitus, erectile dysfunction, hypothyroidism, cardiac enlargement, and joint arthropathy [1].

Majority of the patients affected by HH develop a characteristic arthropathy, described for the first time in 1964 by Schumacher [4] in the metacarpophalangeal (MCP) and proximal interphalangeal (PIF) joints. This characteristic arthropathy can affect different joints; the most affected are the MCP and the ankle joints, followed by the knee and hip [5].

Ankle arthropathy is found in 32–61% of HH patients. It can lead to foot pain during walking, soft tissue swelling, and impairment of range of motion (ROM) of the ankle [4]. Furthermore, it is frequently the first presenting manifestation of the disease [6].

Although ankle arthropathy in patients with HH is well documented in the rheumatology literature, the clinical course and impact of ankle arthropathy in patients with HH are still uncertain, and only a few studies have addressed this topic. Furthermore, there are no specific guidelines for management and treatment, particularly regarding the foot and ankle [7,8]. 

The aim of this review is to give an overview about the current state of knowledge of the diagnosis, management, and treatment of ankle arthropathy in hemochromatosis, as well as to provide clinical and practical guidance to physicians regarding the management and treatment of this disease.

## 2. Epidemiology

The homozygous mutations (C282Y) of the HFE gene are found almost exclusively in white individuals and lead to HFE-related HH (also known as type 1 HH) [9,10].

The prevalence of HFE-related HH is similar in the United States, Europe, and Australia, with approximately 1 case in 200–400 persons [11]. In Italy, the prevalence of the disease may differ between populations of northern origin (1 case in 500 inhabitants) and central-southern origin (less than one case in 2000 inhabitants) [12].

Arthropathy and joint pain have been reported in between 28% and 81% of patients with HH [13], with a slight prevalence in males [14]. The average age of patients with initial joint symptoms is 45.8 ± 13.2 years [15,16]; in particular, symptoms can occur before 30 years of age in men and after menopause in women (due to the protective effect of estrogen and to the iron loss during menstruation and pregnancy) [17,18]. It was further estimated that about 16% of patients with joint involvement undergo joint replacement surgery [15].

Although ankle joint involvement is described in about one out of two patients with HH, as reported by Hamilton et al. [19], there are not many studies in the literature regarding ankle arthropathy in HH. This might suggest that involvement of this joint may be underrecognized in HH or have a late presentation [20].

## 3. Physiopathology

Excess free iron, due to chronic systemic iron overload, could act as a catalyst to produce high levels of reactive oxygen species (ROS) and lead to oxidant-mediated cellular injury [21,22].

Several studies demonstrate that impaired metabolism of iron is associated with bone mass decrease, osteopenia, and osteoporosis, leading to bone weakening, microarchitecture and biomechanical disorders, and an increased risk of fractures [23,24,25].

How iron overload can lead to ankle arthropathy is not well delineated, but clinical studies suggest a direct link between iron overload and joint damage (Table 1). The HFE gene may be acting as a modifier gene, perhaps influencing the pattern of joint involvement or the severity of the chondral damage. Camacho et al. [26] demonstrated that HH is associated with an increased expression of genes related to cartilage degradation (in particular, ADAMTS and MMP-3 [27]) and further with an accelerated development of osteoarthritis (OA). Heiland et al. [28] reported that hemosiderin, an intracellular iron complex, accumulates in the synovial tissue and commonly causes a low–moderate grade of synovitis.

Serum ferritin levels, which reflect the body’s iron stores, also correlate with the severity of subchondral arthropathy [29]. In a 2-year longitudinal observational study, Kennish et al. [30]. found a correlation between serum ferritin levels and a predicted higher risk of severe X-ray disease.

Furthermore, chondrocytes have been shown to express an abnormal HFE protein that leads to dysregulation and iron overload, generation of ROS, and increased apoptosis of chondrocytes [20].

**Table 1:** Pathophysiological mechanisms of HH arthropathy.

Authors; Year	Physiopathology	Clinical Manifestations
Kai et al. [22]; 2021	Increased production of ROS, VEGF, NF-kB	Synovial inflammation and hyperplasia, subchondral bone sclerosis, cartilage damage, OA
Camacho et al. [26]; 2016	Increased iron accumulation in the knee synovial membrane	Cartilage and subchondral bone degradation, OA
Heiland et al. [28]; 2010	Increased hemosiderin, iron, and mononuclear cell infiltration in the synovial space	Formation of microvessels, synovial hyperplasia, cartilage degradation
Kennish et al. [30]; 2014	High ferritin levels in the synovial fluid	Articular damage and slower OA progression

ROS: reactive oxygen species; VEGF: vascular endothelial growth factor; NF-kB: nuclear factor k-activated B cells; OA: osteoarthritis.

## 4. Clinical Presentation and Differential Diagnosis

Among patients with a diagnosis of HH, the most frequently reported symptoms are fatigue and joint pain. Joint pain appears early and, in some cases, can precede the diagnosis of HH by many years [5,15]. Ankle arthropathy was found in 32–61% of HH patients. It can lead to foot pain during walking, soft tissue swelling, and impairment of range of motion (ROM) of the ankle [5]. Schmid et al. [31] described three Caucasian male patients who presented symmetric pain and swelling of the ankles, without a history of trauma; only one of these had a previous diagnosis of HH. Moreover, the presence of non-specific symptoms may delay the correct diagnosis or confuse it with other similar diseases of the ankle, such as rheumatoid arthritis (RA) and primary OA [32].

HH ankle arthropathy differs from RA because arthritis is more common than synovitis, morning stiffness is not a frequent sign, and the arthropathy, degenerative rather than inflammatory, can lead to extensive joint destruction [33,34]. In contrast with primary OA, the onset of HH ankle arthropathy may be early, and it can also appear in young patients. Moreover, the prevalence of primary ankle OA is lower and is usually secondary to trauma [35,36]. Consequently, a correct differential diagnosis is necessary, and common symptoms in HH, such as hepatic fibrosis, diabetes, cardiac disease, and skin pigmentation, may be helpful to obtain a correct diagnosis. Different hematological diseases are associated with iron overload and arthropathy. The symptoms of degenerative arthropathy in HH are, like arthropathy, due to hemophilia or thalassemia [37].

Malignant hematologic diseases can also develop joint manifestations; leukemic arthritis (LA) is a rare symptom and is characterized by inflamed, erythematous, tender joints, especially of the ankle. The most common presentation of LA is acute symmetric polyarthritis, mimicking rheumatoid arthritis; the pathogenesis is due to leukemic infiltration into synovial and peri-synovial tissues [38].

Multiple myeloma arthritis is characterized by inflammatory arthritis with polymorphonuclear leukocytes in synovial fluid, without crystals and sometimes with amyloid infiltration [39]. Also, in cryoglobulinemia, ankles are often affected, generally involving a mild, non-erosive oligo-arthritis, often exacerbated by exposure to cold [39].

## 5. Imaging

Imaging has a central role in the diagnosis and treatment of ankle arthropathy. X-ray is the gold standard, while Magnetic Resonance Imaging (MRI) and Ultrasound (US) are used for the assessment of the surrounding soft tissue.

Standard radiographs of the ankle (antero-posterior and lateral views) are the gold-standard to evaluate structural alterations; bone erosion, joint space narrowing, subchondral sclerosis, and osteophytes are characteristic signs useful in classifying the severity of arthropathy. Ferric salts promote the formation and subsequent deposition of intra-articular calcium pyrophosphate (CPP) crystals [7,40]; joint chondrocalcinosis and subchondral disease are common findings in HH arthropathy and are described in one-third of patients [41,42]. The radiographic changes of the ankle joint are mostly like those of other idiopathic arthropathies (IA). Bone erosion is less frequent than in IA, while the presence of subchondral transparency, chondrocalcinosis, and CPP deposition is higher [16,43,44].

US is used to confirm the inflammatory nature of the arthropathy. In these cases, signs of synovitis, tenosynovitis, and CPP crystal deposition are prominent, often even earlier than the clinical symptoms, underscoring a role of US in predicting deterioration and future clinical evolution [45].

Advanced three-dimensional (3D) computed tomography (CT), and particularly MRI, can be used to provide a more accurate imaging of the number, location, and size of any points of cartilage damage. MRI also allows evaluation of soft tissue damage, synovial hyperplasia, and hemosiderin deposits [46].

Frenzen et al. [47] described heterogeneous inflammatory joint changes, such as erosions, bone marrow edema, and synovitis. Furthermore, they found these changes also in asymptomatic patients, showing that articular changes can be present in the absence of symptoms.

CT provides superior bone resolution and permits multiplanar and 3D reconstruction of data that is useful for assessment of the complex midfoot and hindfoot anatomy [48,49]. For example, high-resolution peripheral quantitative computed tomography (HR-pQCT) can offer advantages over other imaging modalities in detecting fine bone details and cortical deterioration. Jandl et al. [50]. described the use of HR-pQCT in 10 patients affected by HH, and they identified microstructural deterioration and volumetric bone mineralization deficits; specifically, they found pronounced cortical bone loss, with mineralization deficit, reflected by reduced cortical thickness and cortical bone mineral density.

## 6. Treatment

Iron overload is strictly associated with the development of arthropathy; thus, the first line of treatment is based on maintaining body iron homeostasis to prevent arthropathy [22]. When arthropathy is already established, conservative or surgical treatment can be considered to slow down the progression of the disease and treat its symptoms.

It is important to emphasize that there is no one-size-fits-all treatment for all patients; several factors must be first evaluated before deciding whether to opt for surgical or conservative treatment, including the characteristics of the disease, the grade of osteoarthritis, ankle pain, functional status, the patient’s history, and hematologic status.

### 6.1. Conservative Treatment

There are no clinical guidelines for the management of ankle arthropathy, but a conservative approach should always be the first choice in treatment. Although the literature is scarce in studies concerning specific pharmacological or conservative treatments to prevent and treat ankle arthropathy in HH, listed below treatments widely used in HH-related ankle arthropathy. Nevertheless, there is little scientific evidence to support them [35,51,52].

#### 6.1.1. Pharmacological Treatments

- *Iron chelators (ICs)*: although their use remains unclear, and there is no clinical data about the therapeutic effects in HH arthropathy, they are widely used. ICs bind circulating and intracellular iron and allow its elimination through urine or bile. In vitro studies demonstrated that ICs inhibit extracellular matrix (ECM) degradation and reduce iron-induced ROS production and apoptosis of chondrocytes [53,54].
- *Regulators of iron metabolism*: iron-metabolism-related proteins (IMRPs), such as divalent metal transporter 1, deferoxamine, lactoferrin, and ferritin, can affect the functions of chondrocytes, osteoblasts, and osteoclasts [22]. In a study on mice, inhibition of hepcidin (an IMRP) demonstrated increased iron absorption in bone and activity of osteoclasts, with a reduction in that of osteoblasts [22]; therefore, regulation of the metabolism of this protein can be useful in counteracting the effects on bone [55].
- *Antioxidants*: these have been shown in in vitro studies to protect osteoclasts and osteoblasts from oxidative-stress-induced abnormalities resulting from iron overload [53]; furthermore, reducing oxidative stress can prevent chondrocyte damage and cartilage degeneration [54]. For example, N-acetylcysteine (NAC) can protect chondrocytes from oxidative stress caused by Interleukin 1, which is also responsible for chondrocyte apoptosis [53,56].
- *Non-steroidal anti-inflammatory drugs (NSAIDs)*: topical NSAIDs are generally recommended as first-line strategies, and they should be considered as an adjunct to non-pharmacological approaches [57,58]. They are an effective and safe option to control local pain, with results superior to placebos. As an alternative, topical Capsaicin has been shown to be effective [59]. Oral NSAIDs, including cyclo-oxygenase (COX)-2 inhibitors, are recommended at the lowest effective dose, such as 1000 mg/day, and for the shortest possible period. Patients should be carefully monitored, because these cause gastrointestinal, renal, and cardiovascular side effects [60,61].
- *Opiods*: evidence concerning opioid use (such as Tramadol, Morphine, Oxycodone) is weak and not supported by the literature. Currently, their use is contraindicated or accepted only as a third line of treatment [62].
- Additional pharmacological strategies that are not recommended for peripheral joint OA include chondroitin, vitamin D, tricyclic agents, glucosamine, and risedronate [59].

#### 6.1.2. Intra-Articular Injections

Literature shows that intra-articular injections are tolerable and effective in ankle arthropathy and produce a rapid clinical improvement in terms of pain, stiffness, and satisfaction [35,63].

- *Hyaluronic acid (HA)*: one of the most-used substances. The mechanisms of action include anti-inflammatory and chondroprotection effects: HA reduces inflammatory cell migration, stimulates endogenous HA synthesis, and inhibits nociceptors and cartilage-degrading enzymes [51,64]. Sun et al., in their prospective case series of ankle OA followed up for 6 months, showed significant American Orthopaedic Foot and Ankle Society (AOFAS) scale score improvements using three HA intra-articular injections at 1-week intervals [65].
- *Platelet-rich plasma (PRP)*: in the last few years, the application of PRP has become increasingly popular in orthopedic surgery [66,67]. PRP is used for acceleration of bone healing, prevention and treatment of soft-tissue and osseous infection, treatment of acute and chronic tendon or ligament injuries, and pain alleviation of osteoarthritic joints [68,69]. Recent studies have reported that platelet-rich plasma (PRP) therapy seems to be more effective than HA in reducing pain, improving range of motion, and delaying the indication for surgery [70,71,72].
- *Corticosteroids (CSs)*: CSs have anti-inflammatory properties, but their use remains controversial. In fact, CSs also act to inhibit fibroblast proliferation and many protein expressions, causing damaging effects to the joint cartilage or to other structures, such as the plantar fascia [73]. Therefore, they should not be used, or they should be reserved for persistent pain in severe grades of OA, with a maximum of three or four injections a year [74].

#### 6.1.3. Orthoses

Treatment with orthoses and shoe modifications was revealed to be effective in pain reduction by maintaining correct alignment, limiting ankle mobility, and reducing the mechanical load on the ankle [75]. The use of insoles instead remains controversial, and there are no studies confirming their effectiveness. Nevertheless, Tezcan et al. described in a clinical trial the effect of using a lateral wedge in the ankle joint width, finding no clinical deterioration [76]. In advanced cases, the use of a high-top boot, articulated or nonarticulated orthoses, and wedges are important to control pain, motion, and joint stability; they are also useful to postpone surgical approaches [77,78].

#### 6.1.4. Physical Therapy

There is no evidence of the efficacy of physical therapies for ankle arthropathy and arthritis. However, the efficacy of these physical strategies was substantially studied for rheumatoid patients and may also be applied to ankle arthritis originating from causes other than of a rheumatoid nature [64].

- *Strengthening of the musculature and a regular practice of stretching exercises*: can be a beneficial adjunct to the conservative management of ankle arthropathy [79]. Muscle strengthening as well as ankle and foot joint mobilization can prevent stiffness and pain and reduce joint stress and solicitations [80].
- *Electrotherapy*: used to reduce pain and to increase muscle strength and function. In transcutaneous electrical nerve stimulation (TENS), an electrical current is transmitted through electrodes to a specific muscular site of interest to stimulate motor units [79]. TENS either transmits an electric current of high frequency with a low intensity for immediate pain relief or uses high-frequency burst impulses at a low intensity to relieve pain by stimulating pain-carrying fibers [81,82].
- *Thermotherapy*: used to provide short-term pain relief; it includes a cold or hot source or a contrast bath with cold and hot water [79,80,83]. Low-level laser therapy is another modality to treat pain and to improve function in patients with ankle arthropathy. The laser emits a single wavelength of pure light, which causes a photochemical reaction within the cell [84].

### 6.2. Surgical Treatment

Surgical treatment is usually the last option considered. It can include both arthroscopic and open approaches. Surgeons should choose surgical approaches when conservative treatment does not obtain clinical success. Many surgical strategies can be used.

- *Arthroscopic technique*: The arthroscopic approach is reserved for patients who do not respond to other treatments or have already developed early signs of OA [85]. It reduces pain and improves function in patients with clinical signs of anterior impingement and diffuse joint synovitis [51]. In addition, it has been used to associate debridement and synovectomy with arthroscopic bone-marrow-derived cell transplantation (BMDCT) to treat osteochondral lesions [86]. MBDCT includes the production and application of PRP to apply growth factors and a fibrin clot to improve biomaterial implantation and promote regeneration [87].
- *Ankle arthrodesis (AA)*: AA is considered the most useful and successful treatment for end-stage OA (stages 3B and 4 in the Takakura–Tanaka Classification [88]) or after failure of conservative treatment for more than 6 months. It is preferred in young and active patients with high functional requirements. It can be performed either arthroscopically or through open access. Arthroscopic AA has several advantages compared to traditional techniques, including smaller skin incisions, less periosteal stripping, and less soft tissue damage. Two standard anterolateral and anteromedial portals are used, and there is a lower risk of infection [89,90]. Conventional open AA includes different approaches (lateral, anterior, and posterior) and different types of osteosynthesis [35]. The anterior method is performed through a dorsal incision between the tibialis anterior and extensor hallucis longus tendons. Despite being a less invasive approach, it allows good access to both the medial and lateral gutter, spares the fibula and, eventually, allows secondary conversion from AA into Total Ankle Arthroplasty (TAA) [35,37]. The lateral method also provides good surgical site visualization, but in this case, the fibula’s sacrifice is necessary. The posterior approach is the least utilized method; it can be useful in revision, particularly if anterior or lateral soft tissues are poor. The procedure is correctly performed when the ankle is fixed in neutral dorsiflexion, with 0–5° hindfoot valgus and 5–10° external rotation [37].

Types of fixations include screws, plates, retrograde nails, or external fixators [91].

Several studies have compared open and arthroscopic joint fusion; while they offer similar rates of nonunion and fusion times, hospitalization and recovery times strongly favor arthroscopic treatment, though these differences seem to even out after 1 year of follow-up [92].

In the literature, there are no specific studies on AA as a treatment for HH-related severe ankle arthropathy. This may be an interesting and effective solution for surgeons who want to treat young and active patients with this disease.

- *Total ankle arthroplasty (TAA)*: This is an alternative solution to AA in selecting patients with severe OA. Current indications include patients with end-stage OA, sedentary lifestyles, the elderly (above 55 years at present), low functional requirements, and preserved joint mobility [93]. In the literature, there are only a few studies addressing TTA in HH-related ankle arthropathy.

Davies and Saxby [94] described four male patients, with an average age of 60, affected by HH, with bilateral ankle arthropathy. Two patients underwent TAA on one side only, one patient underwent bilateral two-stage TAA (one year apart), and the last patient, previously treated with ankle arthrodesis on one side, underwent TAA on the other side. All patients, at the last follow-up (average of 2.7 years), were satisfied with the treatment and free from pain (Table 2).

Barg et al. [1] conducted a retrospective study on 16 patients affected by HH who underwent TAA, for a total of 21 implants (Table 2). At the end of the follow-up, the tibial and talar components were radiographically stable in all ankles, and the results showed significant improvement in terms of pain improvement (VAS scale), functional scores (ROM and AOFAS scales), and quality of life (SF-36).

## 7. Conclusions

Involvement of the ankle joint in HH is relatively common, and it can lead to severe pain, functional impairment, and bone reabsorption.

There are no distinctive clinical signs or radiological findings to reliably differentiate HH arthropathy from primary OA of the ankle joint; rather, the patient’s clinical history guides and leads to the diagnosis.

The first approach in the treatment of patients with HH arthropathy of the ankle consists of maintaining the iron body homeostasis, even though there are no definitive data on the effectiveness of these treatments. No other conservative treatments are mentioned to improve pain relief. Nowadays, surgery represents the gold standard for the treatment of ankle arthropathy in HH; limited data are available in the literature regarding conservative and surgical treatments.

Treatment should be carried out in a reference center for foot and ankle surgery by a multidisciplinary team that includes expert hematologists and skilled surgeons to guarantee correct disease management.

Several factors, such as pain, patient history, and general and local clinical conditions, must be considered when choosing the most correct treatment.

## Figures and Tables

**Table 2:** Surgical treatment.

Authors; Year	Age; Sex	Involvement; Duration of Symptoms (Years)	Treatment	Follow-Up (Years)	Outcomes
Davies and Saxby [94]; 2006	52; M	Bilateral; 5	One-side TAA	3	Free of pain
	71; M	Bilateral; 10	Two-stage bilateral TAA 1 year later	3	Free of pain
	59; M	Bilateral; 10	- Left ankle arthrodesis (6 years before) - One-side TAA	4	Free of pain
	59; M	Bilateral; 3	One-side TAA	1	Free of pain
Barg et al. [1] 2011	59.5 ± 10.5 (range, 44.4–80.9); 14M, 2F	/	21 total TAA: - 11 unilateral; - 1 simultaneous bilateral; - 4 two-stage bilateral	5.3 (range, 3.1–8.6)	- 4 of the 16 patients were completely pain free, and all patients experienced substantial pain relief. - AOFAS score (46 ± 15 (range, 22–67) preoperatively to 84 ± 6 (range, 74–94) postoperatively - ROM (29.6 ± 10.9 (range, 6–45) preoperatively to 39.3 ± 8.2 (range, 23–56) postoperatively - SF-36 PH 30.5 ± 6.2 to 77.7 ± 4.7 - SF-36 MH 56.4 ± 4.9 to 78.8 ± 2.2

M: male; F: female; TAA: total ankle arthroplasty; AOFAS: American Orthopaedic Foot and Ankle Society score; ROM: range of motion; SF: short-form health survey; PH: physical health; MH: mental health.

## Data Availability

The study data will be available upon request from the corresponding author (email: greco.tommaso@outlook.it).
